# Inferences from the Historical Distribution of Wild and Domesticated Maize Provide Ecological and Evolutionary Insight

**DOI:** 10.1371/journal.pone.0047659

**Published:** 2012-11-14

**Authors:** Matthew B. Hufford, Enrique Martínez-Meyer, Brandon S. Gaut, Luis E. Eguiarte, Maud I. Tenaillon

**Affiliations:** 1 Department of Plant Sciences, University of California Davis, Davis, California, United States of America; 2 Departamento de Zoología, Instituto de Biología, Universidad Nacional Autónoma de Mexico, Mexico DF, Mexico; 3 Department of Ecology and Evolutionary Biology, University of California Irvine, Irvine, California, United States of America; 4 Departamento de Ecología Evolutiva, Instituto de Ecología, Universidad Nacional Autónoma de Mexico, Mexico DF, Mexico; 5 CNRS, UMR 0320/UMR 8120 Génétique Végétale, Gif-sur-Yvette, France; New York State Museum, United States of America

## Abstract

**Background:**

The species *Zea mays* includes both domesticated maize (ssp. *mays*) and its closest wild relatives known as the teosintes. While genetic and archaeological studies have provided a well-established history of *Z. mays* evolution, there is currently minimal description of its current and past distribution. Here, we implemented species distribution modeling using paleoclimatic models of the last interglacial (LI; ∼135,000 BP) and the last glacial maximum (LGM; ∼21,000 BP) to hindcast the distribution of *Zea mays* subspecies over time and to revisit current knowledge of its phylogeography and evolutionary history.

**Methodology/Principal Findings:**

Using a large occurrence data set and the distribution modeling MaxEnt algorithm, we obtained robust present and past species distributions of the two widely distributed teosinte subspecies (ssps. *parviglumis* and *mexicana*) revealing almost perfect complementarity, stable through time, of their occupied distributions. We also investigated the present distributions of primitive maize landraces, which overlapped but were broader than those of the teosintes. Our data reinforced the idea that little historical gene flow has occurred between teosinte subspecies, but maize has served as a genetic bridge between them. We observed an expansion of teosinte habitat from the LI, consistent with population genetic data. Finally, we identified locations potentially serving as refugia for the teosintes throughout epochs of climate change and sites that should be targeted in future collections.

**Conclusion/Significance:**

The restricted and highly contrasting ecological niches of the wild teosintes differ substantially from domesticated maize. Variables determining the distributions of these taxa can inform future considerations of local adaptation and the impacts of climate change. Our assessment of the changing distributions of *Zea mays* taxa over time offers a unique glimpse into the history of maize, highlighting a strategy for the study of domestication that may prove useful for other species.

## Introduction

The teosintes are a group of grass species (Poaceae) from Central America that are traditionally divided into two sections: section *Luxuriantes* and section *Zea* [1]. Section *Zea* contains all diploid, annual species, including cultivated maize, *Zea mays* ssp. *mays,* two widely-distributed teosintes, ssp. *mexicana* (hereafter *mexicana*) and ssp. *parviglumis* (hereafter *parviglumis*) and ssp. *huehuetenangensis* that is endemic to a small region of western Guatemala. *Parviglumis* and *mexicana* are thought to have diverged ca. 60,000 years ago [2], [3] and currently occupy distinct ecological niches: *mexicana* is adapted to the drier and cooler elevations of northern and central Mexico (1600–2700 m) while *parviglumis* is adapted to the warmer, mesic middle elevations of southwestern Mexico (<1800 m). These taxa are therefore geographically well separated, except in the eastern Balsas River Basin of southwest Mexico, where there is evidence of recurrent admixture [4]. This region may constitute a hybrid zone or alternatively may represent the ancestral gene pool from which both *parviglumis* and *mexicana* were derived [4].

Perhaps as a result of adaptation to distinct niches, the wild subspecies exhibit morphological differences: *mexicana* produces larger spikelets and seeds and fewer tassel branches compared to *parviglumis. Mexicana* also has red, hairy leaf sheaths in contrast to the green and glabrous leaf sheaths of *parviglumis* [1], traits thought to be important for adaptation to the cool temperatures of the Mexican highlands [5]. Differentiation at the morphological level is accompanied by genetic divergence [3], [4]. However, it has yet to be resolved whether both subspecies are monophyletic or whether *mexicana* is derived from *parviglumis*. Indeed, *parviglumis* appears paraphyletic in some studies [4], [6]. Both of these teosinte taxa exhibit high nucleotide diversity and population genetic patterns consistent with large effective population sizes and recent population expansions [3].

Phylogeographic studies tend to subdivide *parviglumis* and *mexicana* into distinct regions [4], [6], [7]. *Parviglumis* is often separated into races Balsas and Jalisco, which occur in isolated geographic areas [4], [6], [7], a distinction supported by microsatellite-based analysis of genetic structure [4]. However, at the nucleotide level, population differentiation between these two putative races is not significant [8]. *Mexicana* has often been classified into five geographic regions (Chalco, Nabogame, the Central Plateau, Durango, and Puebla [4], [7]), yet microsatellite analysis has revealed only three distinct genetic clusters: Nabogame, Chalco-Puebla and Durango [4].

Genetic [9], [10] and archaeological [11], [12] data indicate maize was domesticated in the early Holocene (∼9,000 years BP) from *parviglumis* in the lowlands of the Central Balsas. Rapid diffusion of maize outside its center of origin is probable based on the discovery of maize cobs and phytoliths in the Guila Naquitz cave of the Oaxacan highlands (6,250 years BP [13]), in San Andres (Tabasco state; 7,300 BP [14]), and in Paredones and Huaca Prieta (coastal Peru; 6,775–6,504 BP [15]), sites that do not overlap with the current distribution of *parviglumis*. During its diffusion, maize adapted to diverse habitats resulting in a current distribution much wider than its closest relatives with respect to altitude (from 0 to 3,400 m) and latitude (from the central valley of Chile (40° South) to Canada (52° North) [16]). Within Mexico alone landraces encounter extremely diverse environments in terms of mean annual temperature (from 12°C to 29.1°C) and precipitation (from 400 to 3555 mm) [17]. The diversity of growing conditions and cultural preferences in Mexico has led to extensive differentiation of maize races [18], [19]. Four of these landraces—Arrocillo Amarillo, Palomero Toluqueño, Chapalote, and Nal-Tel—are believed to be among the most ancient landraces of maize [20], [21] and likely represent early adaptations during the diffusion of maize from its center of origin.

Perhaps reinforcing local adaptation to diverse environmental conditions, hybridization barriers have been suggested to isolate taxa within *Z. mays*. Pollen flow between maize and teosinte has been shown to occur at low frequency [22] and, while kernels formed on maize ears pollinated by teosinte are fertile, the reverse produces mostly sterile seed. In consequence, when it occurs, gene flow is mostly unidirectional and consists of introgression of teosinte alleles into a maize background. Such introgression was thought to be more common between maize and *parviglumis* than between maize and *mexicana* [4] due to stronger pre-zygotic isolation mechanisms between maize and *mexicana* [23]. However, recent studies seem to contradict this observation [3], [10], [22], [24] suggesting a history of gene flow between *parviglumis* and *mexicana*, between *mexicana* and maize (20% introgression into maize within the Mexican highlands; [10]) and, to a lesser extent, between *parviglumis* and maize.

Clearly, much is known about the evolutionary history and diversity of *Z. mays sensu lato*. However, less is known regarding its ecological history. Previous efforts toward distribution modeling of this species have focused on the potential for gene flow between maize and its wild relatives [25] and the implications for *Z. mays* of projected climate change scenarios [26]. Models of the historical distribution of wild subspecies of *Z. mays* could offer an independent and valuable source of information to complement archaeological and genetic findings regarding domestication, particularly given drastic climatic changes in Mexico near the time of maize domestication. During the Last Glacial Maximum (LGM; ∼21,000 BP) of the Late Pleistocene, temperature is estimated to have been on the order of 4–6°C cooler at lower latitudes in the Americas [27], [28], [29], [30]. The corresponding estimate for precipitation is approximately 10–30% less [29], [31], [32]. The period preceding maize domestication (from 15,500–10,000 BP) saw a dramatic increase in temperature and precipitation in this region [30], [33]. Speleothem records from southwest Mexico, however, suggest climatic shifts back to drier and likely cooler conditions at 10,300 BP and 8,200 BP [34]. The history of abrupt climate shifts near the time of maize domestication complicates inferences regarding the distribution of teosinte at that time, but the overall changes in climate from the LGM to the early Holocene clearly resulted in vegetation shifts in the Central Balsas region with lowland tropical forest replacing herbaceous, cool-adapted flora [27]. It is therefore likely that teosinte currently grows at higher elevation than during the LGM. Relevant and unanswered questions for maize history are the extent to which the distribution of teosinte could have changed during the timeframe of domestication and which portions of the current range of wild relatives reflect refugia of suitable habitat over the course of historical climate change.

In this study, we implemented species distribution modeling, relating ecological variables and species occurrence data - a large compilation of existing passport data from *parviglumis* (316 records), *mexicana* (378 records) and four primitive maize landraces (223 records) – to establish the potential current distribution of the *Z. mays* subspecies (ssps. *mays, parviglumis,* and *mexicana*). In addition, we used paleoclimatic models of the Last Interglacial (LI; ∼135,000 BP; the onset of the Late Pleistocene) and the LGM (near the end of the Late Pleistocene) to hindcast wild *Z. mays* subspecies' past distributions following a non-adaptive model of dispersal. From these distributions we (i) identify the climatic variables primarily determining subspecies' distributions; (ii) revisit current knowledge of domestication and gene flow between subspecies; (iii) identify potential refugia for the wild subspecies over the course of historical climate change; and (iv) suggest locations for future collections.

## Results

We employed ecological niche modeling using MaxEnt software (see Materials and Methods) to obtain past and present distributions of *Z. mays* subspecies. The MaxEnt method is based on machine learning - *i.e.,* it requires both training and testing data sets and models are refined in an iterative process. For these studies, the data consisted of passport occurrence data from each subspecies, separated into training and testing data sets in a 70∶30 ratio (see Materials and Methods for details). The climatic input data for MaxEnt analyses consisted of 19 bioclimatic variables found in the WorldClim data set (www.wordlclim.org; [35]) modeled across three distinct time periods: the present, the LGM (∼21,000 BP), and the LI (∼135,000).

Using these data we obtained probability maps of presence/absence of the subspecies based on consensus among at least five of ten independent runs. The percentage of occurrence data (passport data collected in the field) included in the current predicted distribution maps (six consensus distributions including *mexicana*, *parviglumis* and four maize landraces) was high, ranging from 94.3–100 percent. The predictive power of our MaxEnt models was also confirmed by the Area Under the receiver operating characteristic Curve (AUC). While the AUC estimates the fit of the model to both the training and the testing data, the predictive power of the model is most clearly evidenced by the fit of the model to the testing data. The closer the AUC value is to 1 for the testing data, the better the model performs in predicting the testing sample (see Materials and Methods). We found average AUC values across ten replicates for each taxon ranging from 0.94–0.99 indicating strong performance of our models.

### Current predicted distributions of *parviglumis* and *mexicana*

**Figure 1:**
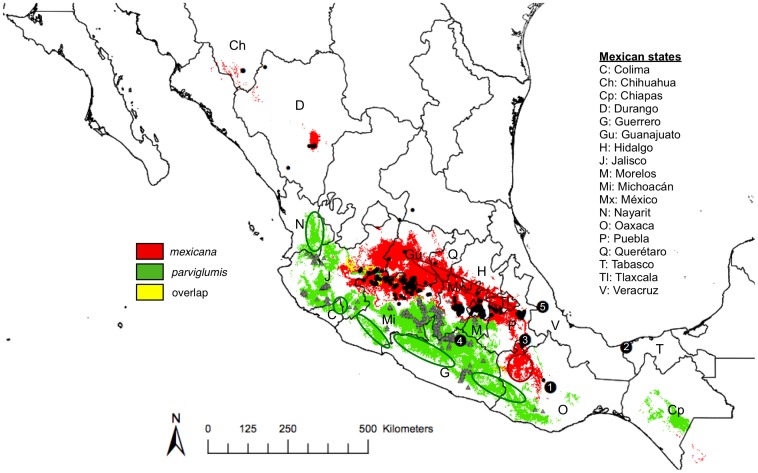
Models of the current distributions of the wild teosintes, *parviglumis* and *mexicana*. Occurrence data used to model the distributions of *parviglumis* (gray triangles) and *mexicana* (black circles). Overlap of the two distributions is indicated in yellow. Numbers denote archaeological evidence of ancient maize: 1) Guila Naquitz (6,250 BP; [13]), 2) San Andres (7,300 BP; [14]), 3) San Marcos (5,500 BP [50]), 4) Iguala (8,700 BP [11]), 5) Veracruz (4,500 BP [51]). Letters represent Mexican states as described in the legend. Circled areas indicate potential gaps in current collections of *parviglumis* (dark green) and *mexicana* (dark red).

Current consensus-predicted distributions of *parviglumis* and *mexicana* and their overlap are shown in Figure 1. The average altitude within the distributions was 1058 m for *parviglumis* and 2105 m for *mexicana* (Table 1). Both distributions were strikingly dissimilar and appeared almost like perfect pieces of a puzzle with small areas of overlap in the eastern portion of the state of Jalisco and in the Central and Eastern Balsas (Figure 1). The complementarity of the distributions clearly derives from ecological differences between the two subspecies (Table 2; Table S1). The climate envelope of *parviglumis* is largely determined by temperature, with the bioclim variables temperature annual range (Table 2; BIO 7) and temperature seasonality (BIO 4) contributing disproportionately to *parviglumis* models, both showing lower values relative to *mexicana*. These observations suggest that *parviglumis* requires a more stable temperature regime than *mexicana*. Precipitation seasonality (BIO 15) also plays a substantial role in *parviglumis*' niche, with higher variability observed relative to *mexicana.* The variables that contribute most to the distribution of *mexicana*, in addition to temperature seasonality and temperature annual range, include the mean temperature of the warmest quarter (BIO 10) and precipitation of the coldest quarter (BIO 19) with *mexicana* showing adaptation to cooler and drier conditions in comparison to *parviglumis*.

**Table 1:** Geographic area, percent current overlap and elevation shifts deduced from the present, last glacial maximum (LGM; based on two General Circulation Models, CCSM3 and MIROC3.2) and last interglacial (LI) potential distributions of *parviglumis* and *mexicana*.

	*parviglumis*				*mexicana*			
	current	LGM-CCSM3	LGM-MIROC3.2	LI	current	LGM-CCSM3	LGM-MIROC3.2	LI
area (km^2^)	59,215	35,844	35,228	10,829	46,295	14,866	44,906	2,567
overlap (% current)	–	60.53	59.49	18.29	–	32.11	97.00	5.54
average elevation (m)	1,058	833	911	524	2,105	1,730	1,871	1,836

**Table 2:** Values and contribution of 19 bioclimatic variables to present distributions of maize landraces and teosintes.

Bioclims^a^	Maize Landraces				Teosintes	
	Arrocillo Amarillo	Chapalote	Nal-Tel	Palomero Toluqueño	*parviglumis*	*mexicana*
BIO 1	154.0	236.8	240.7	147.5	232.7	164.0
BIO 2	135.4	171.8	130.3	153.7	150.5	161.9
BIO 3	65.8	54.8	**67.9**	67.2	66.8	66.5
BIO 4	**1888.2**	5116.3	**1821.6**	*2054.2*	**1725.0**	*2107.4*
BIO 5	*252.9*	*385.9*	333.5	**255.3**	347.0	279.7
BIO 6	49.9	74.1	142.9	27.0	123.0	37.7
BIO 7	**203.1**	311.8	190.7	228.2	*224.0*	*242.0*
BIO 8	164.3	*292.9*	253.0	163.6	235.5	178.6
BIO 9	136.1	231.9	228.7	125.2	232.1	147.6
BIO 10	*175.4*	*298.0*	260.0	**170.5**	255.7	*187.7*
BIO 11	127.4	169.4	214.9	118.5	211.0	133.9
BIO 12	1210.5	630.1	*1263.8*	*905.6*	1113.7	782.2
BIO 13	253.2	174.0	236.9	192.6	249.5	173.6
BIO 14	*24.3*	*3.3*	24.2	8.8	3.7	6.0
BIO 15	78.8	**106.4**	72.5	89.3	*104.8*	95.1
BIO 16	612.9	430.7	615.8	524.7	697.7	469.6
BIO 17	81.6	20.4	84.1	34.3	18.1	25.0
BIO 18	324.5	360.7	364.4	303.9	275.7	232.3
BIO 19	101.9	80.7	109.3	*41.8*	41.5	*31.5*

For each variable, averages across 10 replicates based on occurrence data included in the training set are reported. Significance of the contribution of bioclimatic variables to the present distributions was assessed using three measures: the percent contribution of variables, the permutation importance, and the individual variable contribution (see Materials and Methods). In bold are the variables for which all three measures were ranked among the top-five values and, in italics, variables for which two of three values were in the top-five.

a : Bioclimatic variables defined as: BIO1 = Annual Mean Temperature (°C*10), BIO2 = Mean Diurnal Range (Mean of Monthly Maximum Temperature - Minimum Temperature;°C*10), BIO3 = Isothermality (BIO2/BIO7) (*100), BIO4 = Temperature Seasonality (standard deviation *100), BIO5 = Maximum Temperature of Warmest Month (°C*10), BIO6 = Minimum Temperature of Coldest Month (°C*10), BIO7 = Temperature Annual Range (BIO5–BIO6; °C*10), BIO8 = Mean Temperature of Wettest Quarter (°C*10), BIO9 = Mean Temperature of Driest Quarter (°C*10), BIO10 = Mean Temperature of Warmest Quarter (°C*10), BIO11 = Mean Temperature of Coldest Quarter (°C*10), BIO12 = Annual Precipitation (mm), BIO13 = Precipitation of Wettest Month (mm), BIO14 = Precipitation of Driest Month (mm), BIO15 = Precipitation Seasonality (Coefficient of Variation), BIO16 = Precipitation of Wettest Quarter (mm), BIO17 = Precipitation of Driest Quarter (mm), BIO18 = Precipitation of Warmest Quarter (mm), BIO19 = Precipitation of Coldest Quarter (mm).

Our maps of the potential distributions of *parviglumis* and *mexicana,* when compared to occurrence data, also allow for identification of large regions of suitable habitat from which samples have yet to be collected (Figure 1). For example, there appear to be gaps in collections of *parviglumis* in eastern Nayarit, northeastern Colima, central Michoacán, northwestern Guerrero, and western Oaxaca states. Likewise, future collection efforts for *mexicana* should target a large stretch of suitable habitat found northeast of most current collection sites in the Mexican Central Plateau. This region includes portions of the states of Jalisco, Guanajuato, Querétaro, Hidalgo and Mexico. Additionally, a region in northwestern Oaxaca state appears suitable for *mexicana* yet we lack occurrence data for this region.

### Inferring past distributions of *parviglumis* and *mexicana*

**Figure 2:**
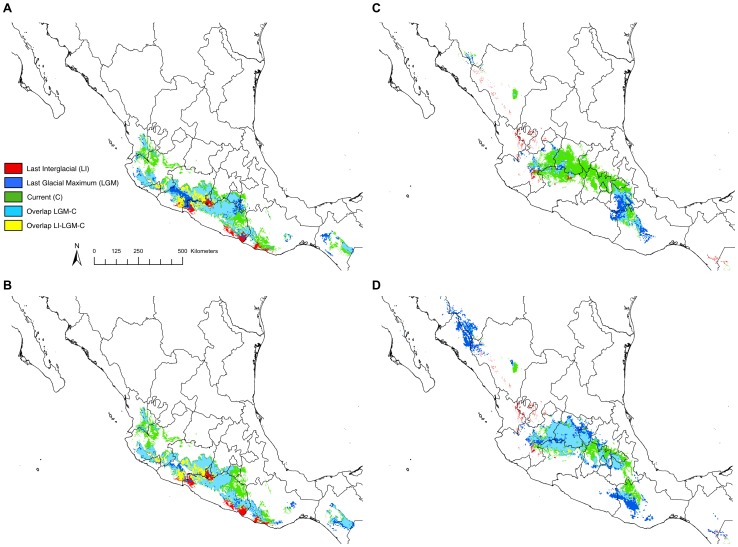
Overlapping models of the changing distributions of the teosintes. The distributions of *parviglumis* (A, CCSM3 and B, MIROC3.2) and *mexicana* (C, CCSM3 and D, MIROC3.2) during the Last Interglacial, Last Glacial Maximum, and currently.

In order to further investigate the domestication and dispersion of maize, we simulated past distributions of the teosinte subspecies using climate models available at two time points: during the last interglacial (LI; ∼135,000 BP) and during the last glacial maximum (LGM; ∼21,000 BP). Available down-scaled environmental data for these time periods include one General Circulation Model (GCM), the Community Climate System Model 3 (CCSM3; [36]), for the LI and two GCMs for the LGM, the CCSM3 and the Model for Interdisciplinary Research on Climate 3.2 (MIROC3.2; [37], [38]). We compared past and present predicted distributions of the two wild subspecies from the LI to the LGM to the present (Figure 2, Figure S1). Quantitatively, the major shift occurred from the LI to the LGM. For example, the LI distributions represented only 18% and 6% of the present distributions of *parviglumis* and *mexicana* respectively, while the LGM distributions represented 60% and 65% (average across both GCMs) of their present distributions (Table 1). Comparison of the distributions also suggests a history of population expansion in both subspecies from the LI to the LGM and a shift toward higher average elevation in *parviglumis* (from 524 m to 872 m) with average elevation remaining relatively constant in *mexicana* (from 1836 m in the LI to 1800 m in the LGM). During this period both models showed *parviglumis* colonizing the Central Balsas region, whereas in *mexicana* the expansion occurred along the Transverse Volcanic Axis and in the state of Oaxaca with a much more dramatic expansion indicated by the MIROC3.2 GCM. Since the LGM, the potential distribution of *parviglumis* has expanded in Nayarit, northern Jalisco and eastern Guerrero states. Likewise, expansions of *mexicana* are supported by both GCMs primarily in Mexico, Tlaxcala, Puebla and Oaxaca states. These expansions also included shifts toward higher average elevation (from an average of 872 m to 1058 m and from 1800 m to 2105 m for *parviglumis* and *mexicana* respectively).

From the overlap of our distributions, we defined geographical zones that remained populated over time from the LI to the present (Figure 2). Such zones define potential refugia. In *parviglumis*, refugia were primarily located in Michoacán and Colima on the border of Jalisco state (Figures 2A & 2B) while in *mexicana* refugia were located primarily in eastern Jalisco and northern Michoacán states (Figures 2C & 2D).

### Predicted distributions for four primitive landraces of maize

**Figure 3:**
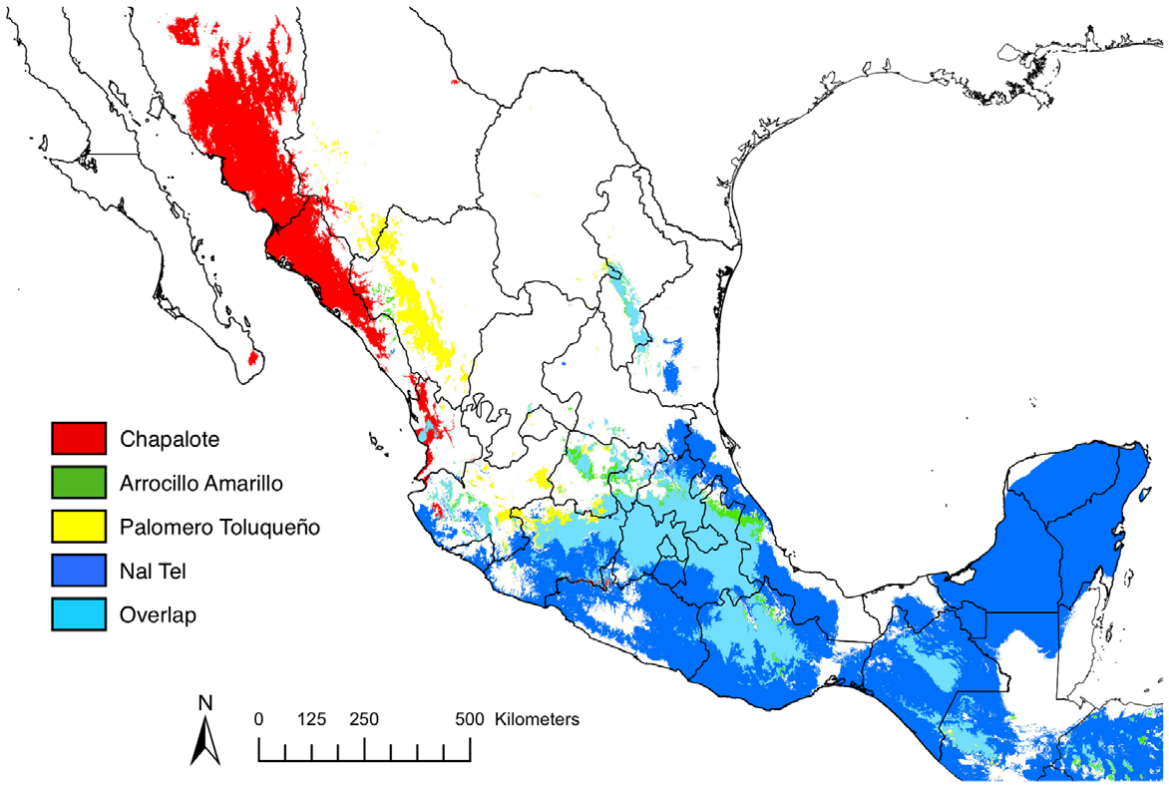
Overlapping models of the current distributions of primitive maize landraces.

In addition to the teosintes, we simulated current predicted distributions of four primitive maize landraces [20], [21], namely Arrocillo Amarillo, Palomero Toluqueño, Chapalote, and Nal-Tel (Figure 3, Figure S2). While Arrocillo Amarillo and Palomero Toluqueño belong to the Central and Northern Highlands group and grow at high elevation, Nal-Tel belongs to the Tropical Dents and Chapalote to the Chapalote group, both being adapted to low elevation [18]. Accordingly, Arrocillo Amarillo and Palomero Toluqueño exhibit similar distributions, confined to the Transverse Volcanic Axis and overlapping, to a large extent, with the distribution of the *mexicana* subspecies. The distribution of Chapalote spans the warm coastal regions of the states of Nayarit, Sinaloa and Sonora, in the north of Mexico while Nal-Tel exhibits the broadest distribution of all landraces, covering most of southern Mexico from portions of the Transverse Volcanic Axis to the tropical climate of the Yucatan Peninsula. The potential distribution of these primitive landraces extends beyond those of the wild subspecies (Figure S3) supporting previous observations [14] that maize adaptation to novel climes in Mexico occurred rapidly subsequent to its initial domestication. As found in the teosintes, temperature seasonality (BIO4; Table S1) is an important contributor to three of the four landrace distributions (Arrocillo Amarillo, Nal-Tel, and Palomero Toluqueño) with these races exhibiting intermediate values between *parviglumis* and *mexicana* for this variable (Table 2). However, each landrace also has unique bioclimatic variables most important in determining its distribution (Table 2). For example, the distribution of Palomero Toluqueño is determined largely by the maximum temperature of the warmest month (BIO 5) and the mean temperature of the warmest quarter (BIO 10), showing marked adaptation of this landrace to cool conditions. The distribution of Nal-Tel, like *parviglumis,* is confined largely to regions with high isothermality (BIO 3). Chapalote is the only *Zea* taxon we investigated with a distribution based primarily on precipitation, with precipitation seasonality (BIO 15) driving its distribution. Finally, annual temperature range (BIO 7) was most important for determining the distribution of Arrocillo Amarillo, which had a somewhat more narrow temperature range and cooler average temperatures when compared to other landraces.

## Discussion

The history of maize domestication has received much attention in the literature from evolutionists and paleobotanists (for a review, [39]). In parallel, a handful of studies on teosinte and maize landrace phylogeography have been published among which two contrast samples from cultivated maize and its closest wild relatives [9], [10]. However, the current potential distributions of these subspecies have not been well described and no inference has been made on their distributions around the time of domestication. In this paper, we used niche modeling, based on the most complete occurrence data for *parviglumis* and *mexicana* assembled to date, in order to predict past and present distributions of these maize relatives. Additionally we described present distributions of four primitive maize races. We discuss our results in light of current knowledge of maize history, revisiting hypotheses previously formulated regarding its domestication and migration. We believe that this type of approach could be applied to other crops, bringing new insight to their domestication and evolution.

Our occurrence data set allowed us to model the ecological niche of each taxon and then hindcast distributions based on this niche using models of past climate from two time points, the LI (∼135,000 BP) and the LGM (∼21,000 BP) for the wild relatives. Based on nucleotide sequence, current estimates of the time of divergence between the two wild subspecies, *mexicana* and *parviglumis*, is ∼60,000 BP [3]. The interpretation of the older time point (∼135,000 BP) therefore remains difficult. Whether the distribution of the ancestral species resembled that of *parviglumis*, *mexicana*, or was different from both is unknown. We nevertheless were able to make four interesting observations.

First, patterns of expansion from the LI and LGM to the present in *parviglumis* and *mexicana* are consistent with sequence data in *parviglumis* that revealed deviations from neutral equilibrium expectations (*i.e.,* significant and positive values of the exponential growth parameter) in three populations from the Balsas and two populations from Jalisco [8]. These patterns of expansion are also consistent with previous observations of an excess of low frequency polymorphisms (*i.e.,* an excess of singletons and negative values of Tajima's D statistic) in both *mexicana* and *parviglumis* [3], [40].

Second, elements of the phylogeography and history of migration of the wild subspecies remain to be resolved. While Moeller *et al.* [8] have detected asymmetric gene flow between Balsas and Jalisco populations with stronger migration from Jalisco to the Balsas, others have indicated that Balsas populations were basal to Jalisco [4] and that the Balsas region may have served as refugia during the LGM [6]. This pattern has been interpreted as evidence for the emergence of Jalisco populations from the Balsas. That Balsas populations are basal to Jalisco had in fact very little if no statistical support [9]; according to our distribution models (Figure 2a), the most stable habitat for *parviglumis* from the LI to the present is located mainly in the states of Michoacán and Colima near the border of Jalisco. In fact, populations sampled in Jalisco state in [9] overlap with our potential refugia. Our data suggest that Jalisco populations may have colonized the Balsas and stress the importance of sampling additional populations in Colima and Michoacán states for inclusion in future phylogeographic studies. We also note that refugia for *mexicana* were located in the Central Plateau (Figure 2b), which seems to corroborate the scattering of Central Plateau accessions throughout other phylogenetic groups (*e.g.,* Nabogame and Durango) [4]. Future studies will benefit from a more extensive sampling in northeast Jalisco and Guanajuato.

Third, concerning historical and recent gene flow between the two wild subspecies, our data offer little support for distribution overlap in present and ancient times, except perhaps in eastern Jalisco and the Central and Eastern Balsas (Figure 1), corroborating observations [4] of several admixed individuals in these regions. However, a scenario with continuous gene flow between *mexicana* and *parviglumis* seems unlikely, except, perhaps, in very limited regions of potential hybridization or due to long-distance dispersal, or, as [3], [10] have suggested, unless maize has served as a genetic bridge between the two wild subspecies. This last scenario is supported by the distribution of primitive landraces largely overlapping those of both wild subspecies (Figure 3).

Finally, rapid dispersion of maize from its center of origin is likely subsequent to domestication. Current distributions of the wild subspecies are similar to the distributions obtained from the LGM (Figure 2) suggesting that there has been little change in the subspecies' ranges from the time of domestication to the present. It is striking then that, among the five Mexican archaeological sites where maize cobs have been found, four (San Andres, San Marcos, Veracruz and Guila-Naquitz) do not overlap with the present modeled distributions of the maize progenitor, *parviglumis* (Figure 1). This seems to confirm previous findings that maize spread rapidly from its center of origin, for example, reaching the state of Tabasco by 7,300 BP [14] and coastal Peru as early as 6,775 BP [15].

The main pitfall of niche modeling is that it ignores species adaptation over time and predicts past distributions by modulating the current species range according to climate records. While this method potentially overlooks important niche evolution, previous comparison of niche models to pollen records of broadly distributed North American species from both the LGM and the present suggests general niche conservatism over this timeframe [41]. Despite potential caveats regarding the non-adaptive nature of niche modelling, the method does provide a list of variables that define the species' range as it is currently adapted. For *Z. mays*, our analyses indicate that each subspecies has contrasted ecological requirements with temperature seasonality and temperature in general appearing as key parameters both in cultivated maize and the teosintes. These requirements could serve as a guide for studying patterns of local adaptation. For instance, pathways involved in cold tolerance or flowering time may exhibit signatures of selection along temperature gradients in the wild taxa, pointing to alleles of agronomic interest that could enhance maize adaptation to cooler climates. In this respect, the *mexicana* gene pool has been largely under-exploited. Finally, our results are in agreement with previous findings [26] in indicating that future climate changes, particularly changes in temperature, may severely impact the distribution of *Z. mays* and stress the importance of characterizing and preserving existing genetic resources.

## Materials and Methods

Geographic distributions for the teosintes and maize landraces were inferred via ecological niche modeling (ENM). ENM is a correlative, static approach that focuses on identifying non-random relationships between occurrence data of a species and a set of environmental variables across the landscape in order to reconstruct the Hutchinsonian multidimensional niche [42] and produce a map resembling its potential geographic distribution [43]. To accomplish this goal, we carried out three steps: (1) input data preparation, (2) niche modeling, and (3) *post hoc* analyses.

### Input data preparation

Two sources of primary data are needed in the niche modeling processes: (a) occurrence data for a species and (b) environmental information in the form of GIS raster layers [44]. Occurrence data for the two teosinte species and the four maize landraces were obtained under an agreement with the Mexican Commission of Biodiversity (Conabio), which has devoted the last five years to compiling and organizing a database with all available data on maize and its wild relatives across the country. A total of 917 spatially unique records were compiled, 316 for *mexicana* and 378 for *parviglumis*, 89 for Arrocillo Amarillo, 19 for Chapalote, 86 for Nal-Tel, and 29 for Palomero Toluqueño. These four landraces are believed to be indigenous to Mexico and among the most ancient of all landraces [20], [21]. According to the classification of [18], two landraces (Arrocillo Amarillo and Palomero Toluqueño) belong to the Central and Northern Highlands group, and grow at high elevation, Nal-Tel belongs to the Tropical Dents and Chapalote belongs to the Chapalote group. Both Nal-Tel and Chapalote are adapted to low elevation.

Environmental information for the present, the LGM, and the LI was drawn from 19 bioclimatic variables found in the WorldClim data set (www.wordlclim.org; [35]). These data largely reflect seasonal and annual trends of temperature and precipitation. Current climate is represented by interpolation of observed data over the period 1950–2000. Paleoclimatic reconstructions for the LGM were statistically downscaled from two different GCMs of the Paleoclimate Modeling Intercomparison Project Phase II database (PMIP 2), the CCSM3 [36] and the MIROC3.2 [37], [38] GCMs, whereas LI data were available only from the CCSM3 GCM. Environmental layers for the present and the LI had a spatial resolution of 0.01 decimal degrees (∼1 km^2^) whereas the LGM was at a resolution of 0.04 decimal degrees (∼17 km^2^).

### Niche modeling

Models were generated with the widely used MaxEnt software [45]. MaxEnt estimates the ecological niche of species by constructing density curves of the sample occurrence data in every environmental variable contrasted against equivalent density curves of background samples; a function is then fitted to this relationship constrained by the mean values of variables and following the maximum entropy principle, i.e., maximizing uniformity of the curve [45]. Finally, MaxEnt values are log-transformed and a map representing probabilities of occurrence is produced [46], [47]. To run the models, we split the occurrence data in a 70∶30 proportion of training/testing data and followed recommendations by [47] regarding parameterization of the interface. We repeated this process ten times resampling training and testing data, with the aim of realizing model stability, because highly similar models under different combinations of training/testing data is an indication of niche conservatism, a mandatory condition for transferring niche models through time [48]. Final probability maps were converted to binary (i.e., presence/absence), using a threshold of the probability value in which omission rate of both training and testing data was under 10% and prevalence (i.e., area predicted as present) was smallest, to reduce commission error. As typically practiced, niche models produced under current climatic conditions were projected onto LGM and LI climatic scenarios using MaxEnt [48]. Finally, models for the present were validated with the widely-used method of the area under the curve (AUC) of the receiver-operating characteristic (ROC) curve [49], which evaluates the capacity of the models to predict presence and absence at different thresholds.

### *Post hoc* analyses

Agreement among the ten binary maps produced per taxon with MaxEnt was determined through map algebra in a GIS. A final consensus map with values ranging between 0–10 was obtained, in which 0 represents areas where all maps predicted the absence of the species, 1 indicates areas where one out of ten maps predicted the presence of the species, and so on until 10, which represents areas where all ten models predicted the presence of the species. Our final distributions required agreement values ≥5 across the ten models. These procedures were carried out for present, LGM and LI distributions.

Contribution of the bioclim variables to the niche models of the various taxa was determined based on multiple summary statistics generated during the MaxEnt analysis: 1) Percent Contribution: as the model is trained, MaxEnt modifies coefficients of the different bioclim variables and records which variable contributes to improving the model. These contributions are converted to percentages at the end of the training process; 2) Permutation Importance: each variable's value in the final model is permuted and the loss in fit to the training data indicates how heavily the model depends on that individual variable; 3) Individual Variable Contribution: each variable is used individually to build a species niche model and the fit of the model to the data is gauged for each variable separately. These summary statistics were averaged across the ten independent MaxEnt runs. We considered those variables that were consistently important (ranked in the top five variables) across these summary statistics as the best candidates for niche determination.

## Supporting Information

Table S1 **Contribution of individual bioclimatic variables to present niche determination of** ***Z. mays*** **taxa.**
(DOCX)

Figure S1 **Individual teosinte distributions over time.** Individual distributions of *parviglumis* (top) and *mexicana* (bottom) during the Last Interglacial, Last Glacial Maximum (CCSM3) and currently.
(TIFF)

Figure S2 **Individual current distributions of primitive maize landraces.** Individual distributions for Chapalote (A), Palomero Toluqueño (B), Arrocillo Amarillo (C) and Nal-Tel (D).
(TIFF)

Figure S3 **Relative current distributions of teosinte versus maize landraces.**
(TIFF)
