# Supplementary material for: Inferences from the Historical Distribution of Wild and Domesticated Maize Provide Ecological and Evolutionary Insight
Source: PLoS One. 2012 Nov 14;7(11):e47659. doi: 10.1371/journal.pone.0047659 (PMC3498274; doi:10.1371/journal.pone.0047659)
Supplement: Table S1 — Contribution of individual bioclimatic variables to present niche determination of Z. mays taxa. (DOCX) [file pone.0047659.s001.docx]

**Table S1.** Contribution of individual bioclimatic variables to present niche determination of *Z. mays* taxa.

|  | **Maize Landraces** | | | | | | | | | | | | **Teosintes** | | | | | |
| --- | --- | --- | --- | --- | --- | --- | --- | --- | --- | --- | --- | --- | --- | --- | --- | --- | --- | --- |
|  | **Arrocillo Amarillo** | | | **Chapalote** | | | **Nal-Tel** | | | **Palomero Toluqueño** | | | ***parviglumis*** | | | ***mexicana*** | | |
| **Variable** | PC | PI | IC | PC | PI | IC | PC | PI | IC | PC | PI | IC | PC | PI | IC | PC | PI | IC |
| **BIO 1** | 0.06 | 0.19 | 0.83 | 0.06 | 0.00 | 0.51 | 5.64 | 0.26 | 0.93 | 0.11 | 0.00 | 1.33 | 0.23 | 0.20 | 0.62 | 0.05 | 0.00 | 0.69 |
| **BIO 2** | 0.28 | 0.71 | 0.10 | 10.33 | 1.27 | 0.46 | 3.65 | 3.22 | 0.28 | 0.04 | 0.00 | 0.30 | 4.82 | 4.11 | 0.69 | 6.81 | 1.23 | 0.81 |
| **BIO 3** | 0.00 | 0.00 | 0.87 | 0.76 | 0.16 | 0.01 | **9.54** | **6.33** | **1.09** | 0.21 | 0.24 | 1.07 | 4.30 | 0.45 | 1.38 | 0.11 | 0.49 | 1.18 |
| **BIO 4** | **28.76** | **68.08** | **1.46** | 0.00 | 0.00 | 0.00 | **48.33** | **40.36** | **1.20** | *15.64* | *1.90* | *1.22* | **29.55** | **19.94** | **1.46** | *17.16* | *3.01* | *1.42* |
| **BIO 5** | *14.61* | *0.27* | *2.17* | *15.38* | *3.00* | *1.25* | 0 | 0.06 | 0.04 | **5.01** | **30.41** | **2.26** | 0.16 | 0.53 | 0.15 | 0.10 | 0.18 | 1.33 |
| **BIO 6** | 0.18 | 4.46 | 0.41 | 0.36 | 0.03 | 0.09 | 2.28 | 1.6 | 1.14 | 0.26 | 0.61 | 0.34 | 5.28 | 2.88 | 1.47 | 6.61 | 38.14 | 0.88 |
| **BIO 7** | **7.70** | **12.49** | **1.43** | 0.13 | 0.00 | 0.05 | 4.6 | 1.84 | 1.32 | 0.13 | 0.00 | 1.14 | *9.14* | *1.19* | *1.48* | *3.33* | *3.19* | *1.55* |
| **BIO 8** | 0.14 | 0.03 | 1.44 | *33.86* | *0.59* | *1.38* | 1.77 | 6.88 | 0.44 | 0.25 | 0.08 | 1.70 | 0.38 | 3.96 | 0.33 | 0.04 | 0.73 | 1.30 |
| **BIO 9** | 4.05 | 0.93 | 0.74 | 0.00 | 0.00 | 0.42 | 0.23 | 0.13 | 0.49 | 3.26 | 12.57 | 1.13 | 1.17 | 11.14 | 0.60 | 0.31 | 1.76 | 0.90 |
| **BIO 10** | *32.65* | *0.00* | *1.90* | *0.83* | *9.42* | *1.05* | 0.02 | 0.06 | 0.19 | **59.21** | **6.90** | **2.30** | 0.04 | 0.06 | 0.19 | *34.71* | *0.95* | *1.61* |
| **BIO 11** | 0.01 | 0.00 | 0.46 | 0.00 | 0.00 | 0.15 | 0.26 | 0.17 | 1.13 | 0.00 | 0.03 | 0.39 | 9.63 | 0.83 | 1.22 | 0.97 | 1.44 | 0.90 |
| **BIO 12** | 0.00 | 0.00 | 0.37 | 0.01 | 0.05 | 0.14 | *11.2* | *17.66* | *0.82* | *3.32* | *8.45* | *0.55* | 3.61 | 3.10 | 1.01 | 8.95 | 1.52 | 1.19 |
| **BIO 13** | 0.80 | 0.86 | 0.51 | 0.05 | 0.00 | 0.02 | 0.35 | 1.48 | 0.94 | 0.00 | 0.00 | 0.38 | 0.01 | 0.07 | 1.19 | 0.12 | 0.22 | 0.74 |
| **BIO 14** | *8.58* | *6.32* | *0.33* | *1.79* | *61.27* | *0.75* | 5.89 | 2.79 | 0.44 | 1.29 | 1.19 | 0.57 | 0.26 | 42.76 | 0.86 | 0.57 | 28.80 | 1.04 |
| **BIO 15** | 0.29 | 0.17 | 0.73 | **30.25** | **8.80** | **1.06** | 1.99 | 4.33 | 0.25 | 0.49 | 0.89 | 0.60 | *20.50* | *3.51* | *1.30* | 0.31 | 0.23 | 0.91 |
| **BIO 16** | 0.01 | 0.00 | 0.47 | 0.03 | 0.10 | 0.01 | 1.14 | 3.33 | 0.87 | 1.43 | 0.98 | 0.47 | 3.04 | 0.48 | 1.29 | 1.52 | 8.89 | 0.79 |
| **BIO 17** | 0.15 | 0.16 | 0.34 | 0.41 | 8.56 | 0.51 | 0.27 | 1.17 | 0.46 | 0.02 | 0.00 | 0.48 | 5.29 | 2.72 | 0.88 | 0.34 | 1.89 | 1.10 |
| **BIO 18** | 0.77 | 0.65 | 0.26 | 3.24 | 0.77 | 0.09 | 0.43 | 0.55 | 0.53 | 0.62 | 1.04 | 0.31 | 2.04 | 1.70 | 0.36 | 1.89 | 1.13 | 0.59 |
| **BIO 19** | 0.96 | 4.60 | 0.12 | 2.51 | 5.97 | 0.24 | 2.36 | 7.73 | 0.37 | *8.72* | *34.68* | *0.42* | 0.57 | 0.36 | 0.52 | *16.14* | *6.14* | *1.02* |

Significance of the contribution of bioclimatic variables to the present distributions was assessed using three measures: the percent contribution of variables (PC), the permutation importance (PI), and the individual variable contribution (IC; only ranks of top five are listed). In bold are the variables for which all three measures were ranked among the top-five values and, in italics, variables for which two of three values were in the top-five.

^a^: Bioclimatic variables defined as: BIO1 = Annual Mean Temperature (°C*10), BIO2 = Mean Diurnal Range (Mean of Monthly Maximum Temperature - Minimum Temperature;°C*10), BIO3 = Isothermality (BIO2/BIO7) (*100), BIO4 = Temperature Seasonality (standard deviation *100), BIO5 = Maximum Temperature of Warmest Month (°C*10), BIO6 = Minimum Temperature of Coldest Month (°C*10), BIO7 = Temperature Annual Range (BIO5-BIO6; °C*10), BIO8 = Mean Temperature of Wettest Quarter (°C*10), BIO9 = Mean Temperature of Driest Quarter (°C*10), BIO10 = Mean Temperature of Warmest Quarter (°C*10), BIO11 = Mean Temperature of Coldest Quarter (°C*10), BIO12 = Annual Precipitation (mm), BIO13 = Precipitation of Wettest Month (mm), BIO14 = Precipitation of Driest Month (mm), BIO15 = Precipitation Seasonality (Coefficient of Variation), BIO16 = Precipitation of Wettest Quarter (mm), BIO17 = Precipitation of Driest Quarter (mm), BIO18 = Precipitation of Warmest Quarter (mm), BIO19 = Precipitation of Coldest Quarter (mm)
