# Supplementary figures and images for: Inferences from the Historical Distribution of Wild and Domesticated Maize Provide Ecological and Evolutionary Insight
Source: PLoS One. 2012 Nov 14;7(11):e47659. doi: 10.1371/journal.pone.0047659 (PMC3498274; doi:10.1371/journal.pone.0047659)

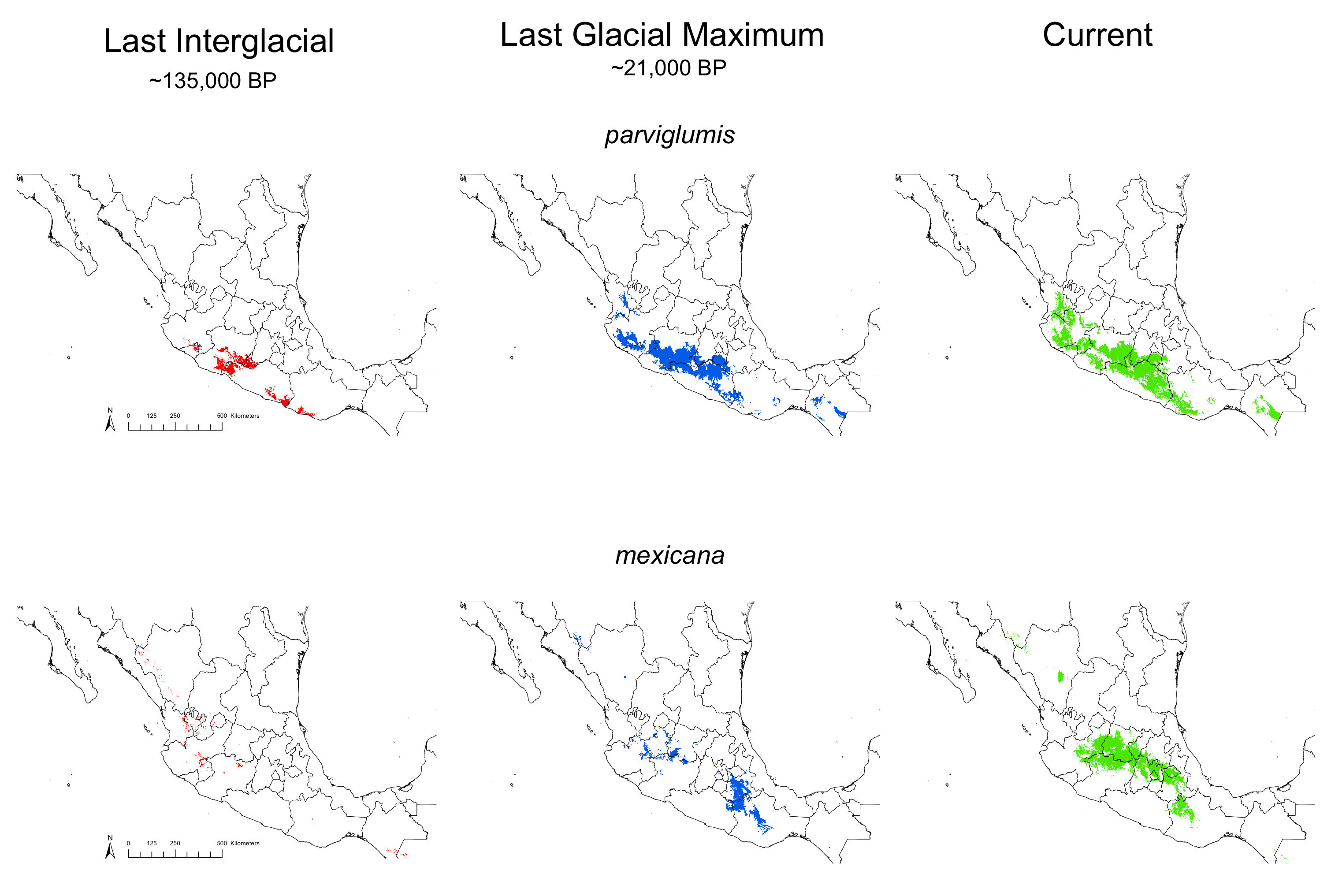

Supplement: Figure S1 — Individual teosinte distributions over time. Individual distributions of parviglumis (top) and mexicana (bottom) during the Last Interglacial, Last Glacial Maximum (CCSM3) and currently. (TIFF) [file pone.0047659.s002.tiff]

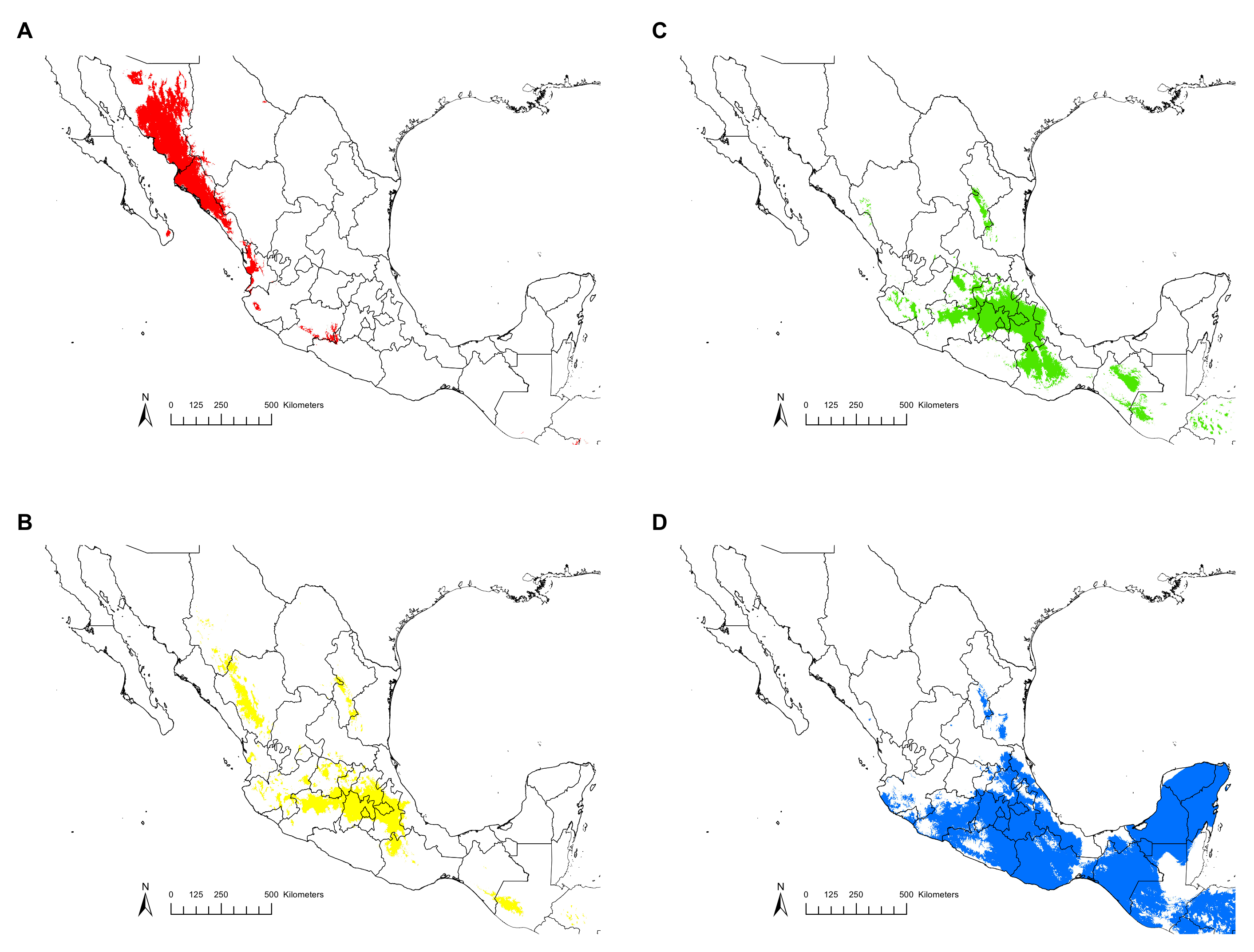

Supplement: Figure S2 — Individual current distributions of primitive maize landraces. Individual distributions for Chapalote (A), Palomero Toluqueño (B), Arrocillo Amarillo (C) and Nal-Tel (D). (TIFF) [file pone.0047659.s003.tiff]

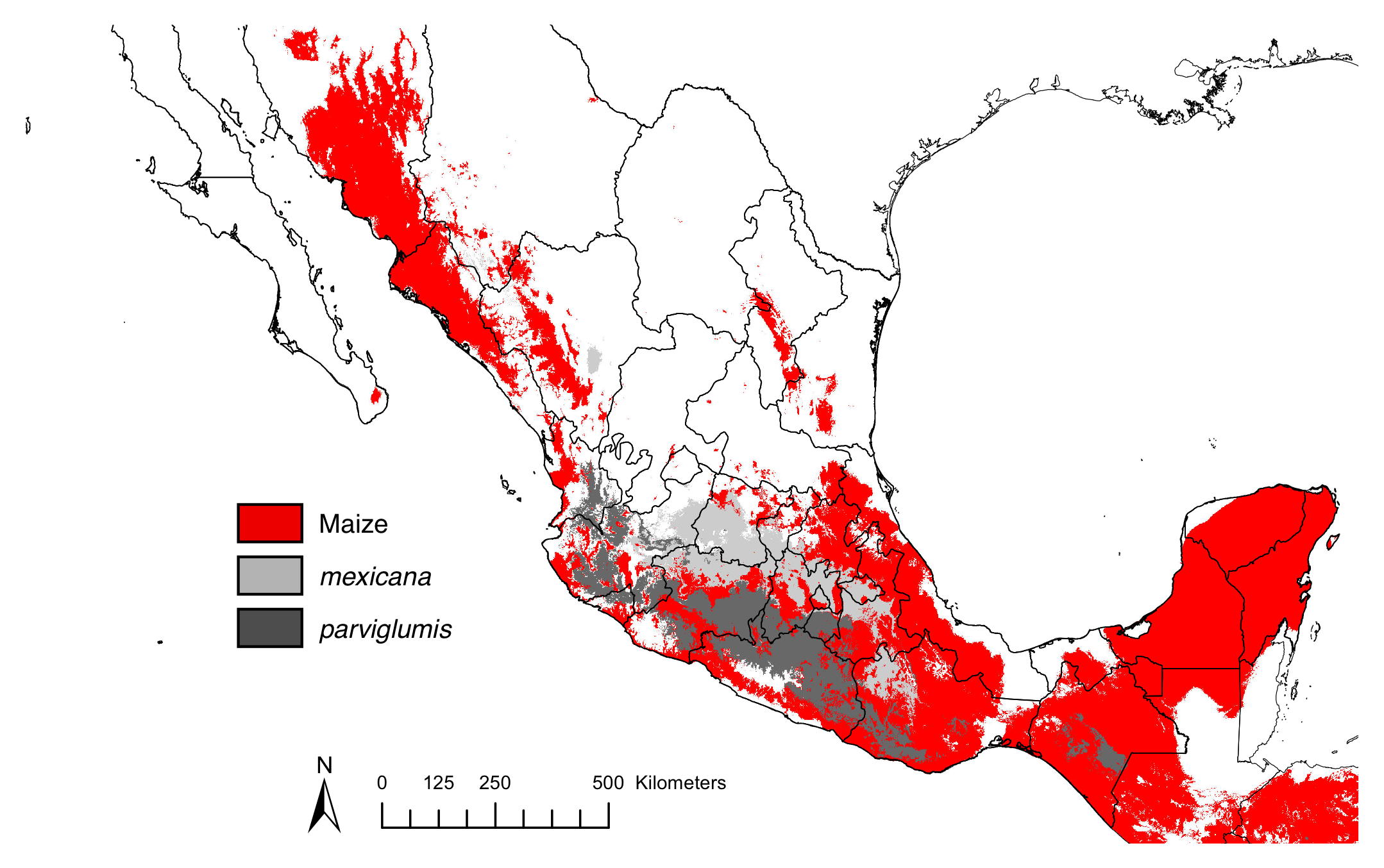

Supplement: Figure S3 — Relative current distributions of teosinte versus maize landraces. (TIFF) [file pone.0047659.s004.tiff]
